# Polyphenol Powders from *Ginkgo biloba* L. and *Clitoria ternatea* L.: Influence of Drying Techniques and Carriers on Antioxidant Capacity and Polyphenol Release Profiles

**DOI:** 10.3390/antiox14121447

**Published:** 2025-12-01

**Authors:** Alicja Kucharska-Guzik, Jessica Brzezowska, Klaudia Masztalerz, Mariusz Nejman, Łukasz Guzik, Anna Michalska-Ciechanowska

**Affiliations:** 1 Laboratorium Analiz Chemicznych Spark-Lab Sp. z o.o., Aleja Zwycięstwa 96/98, 81-451 Gdynia, Poland; lukasz.guzik@spark-lab.pl; 2 Department of Fruit, Vegetable and Plant Nutraceutical Technology, The Faculty of Biotechnology and Food Science, Wrocław University of Environmental and Life Sciences, Chełmońskiego 37 Str., 51-630 Wrocław, Poland; jessica.brzezowska@upwr.edu.pl; 3 Institute of Agricultural Engineering, Wrocław University of Environmental and Life Sciences, Chełmońskiego 37 Str., 51-630 Wrocław, Poland; klaudia.masztalerz@upwr.edu.pl (K.M.); mariusz.nejman@upwr.edu.pl (M.N.)

**Keywords:** plant extracts, inulin, maltodextrin, *Ginkgo biloba* L. powder, *Clitoria ternatea* L. powder, polyphenol characteristic, dissolution study

## Abstract

This study evaluated the impact of the drying and carrier type on the physicochemical and functional properties of *Ginkgo biloba* L. and *Clitoria ternatea* L. extracts and their blends, at ratios of 1:1, 1:2, and 2:1 (*w*/*w*). Extracts were obtained using water as a green solvent and dried by freeze− or spray drying with maltodextrin or inulin. Powders were characterized for moisture content, water activity, color, polyphenol composition (HPLC–MS/MS), and antioxidant capacity (Folin–Ciocalteu, TEAC ABTS, FRAP). Spray−dried samples exhibited lower moisture content and water activity, while freeze-drying ensured higher polyphenol levels. The 1:1 (*w*/*w*) blend with inulin showed the most favorable balance between stability and antioxidant capacity, indicating synergistic effects. This formulation was selected for pilot-scale processing and encapsulated into hard gelatin capsules, which demonstrated rapid polyphenols release under simulated gastric conditions. The findings highlight the potential of spray-dried polyphenol-rich powders as standardized ingredients for immediate-release dietary supplements.

## 1. Introduction

Dietary supplements derived from plant-based materials rich in polyphenols have gained increasing popularity among consumers due to their well-documented health benefits. Polyphenol-rich supplementation has been associated with improved cardiovascular function, reduced risk of neurodegenerative diseases, and enhanced immune response. Furthermore, their therapeutic potential includes antimicrobial properties and anti-inflammatory effects, contributing to improved health state [1].

Among polyphenol-rich plants, *Ginkgo biloba* L. is particularly notable for its high content of flavonoids and terpenoids, known for their neuroprotective and circulation-enhancing effects [2]. Leaf extracts of *Ginkgo biloba* L. have long been used in phytotherapy to alleviate mild cognitive impairment, concentration difficulties, and symptoms of dementia [3], making it one of the most commercialized medicinal plants worldwide. The plant’s antioxidant capacity is primarily attributed to polyphenolic compounds such as rutin, myricetin, quercetin, quercitrin, luteolin, kaempferol, apigenin, and isorhamnetin [4]. Despite the chemical changes occurring during digestion, Oliveira et al. [5] demonstrated that *Ginkgo biloba* L. extracts retain antioxidant and antigenotoxic activity in vitro. Additionally, *Ginkgo biloba* L. seeds meet the criteria for functional foods, providing not only essential nutrients but also health-promoting components, including micronutrients, tocopherols, fatty acids, and terpenoids [6]. As an example, the enrichment of orange juice with *Ginkgo biloba* L. seed extract has also been shown to increase vitamin C and total polyphenol content, with enhanced antioxidant capacity preserved even after extended storage [7].

*Clitoria ternatea* L. is another plant gaining attention for its polyphenol content, particularly anthocyanins, which contribute to oxidative stress protection [8]. Native to Asia, its vividly colored petals are utilized in the food industry as a natural dye. Siti Azima et al. [9] confirmed both its antioxidant capacity and suitability for use as a natural food-grade antioxidant. A patent filed in 2018 described the use of *Clitoria ternatea* L. flower extract in muffins to provide natural coloring with consumer safety [10]. Zainol et al. [11] further validated the relatively high polyphenol content and antioxidant potential of ethanolic extracts, recommending its application in pharmaceutical and food sectors. In 2022, the FDA approved *Clitoria ternatea* L. extract from Sensient (Germany) as a natural food coloring agent for a wide range of products, including beverages, dairy, confectionery, and snacks. As the first plant-based source of natural blue coloring, it overcomes the long-standing challenge of achieving stable natural blue hues [12]. The described extraction method is efficient, economical, and environmentally friendly, offering both functional and health benefits.

The hypothesis of this study assumed that the combination of two polyphenol-rich plant materials, *Ginkgo biloba* L. and *Clitoria ternatea* L., in various proportions, together with appropriate formulation using selected carriers and drying techniques (freeze-drying and spray drying), would enable the development of stable powders exhibiting high antioxidant capacity, favorable physicochemical properties, and efficient release profiles of bioactive compounds. *Ginkgo biloba* L. provides flavonol glycosides with well-documented neuroprotective and circulatory benefits [3], whereas *Clitoria ternatea* L. offers anthocyanins (ternatins) with strong antioxidant and color-stabilizing properties [13]. Ratios of 1:1, 1:2, and 2:1 (*w*/*w*) were selected to investigate potential additive and synergistic interactions between flavonol- and anthocyanin-rich matrices, in line with previous reports on polyphenol–polyphenol synergy [14,15]. The objective of the study was to develop and compare the functional properties of powdered aqueous extracts obtained from *Ginkgo biloba* L., *Clitoria ternatea* L., and their mixes in ratios of 1:1, 1:2, and 2:1 (*w*/*w)*, prepared using two drying techniques and formulated with inulin or maltodextrin as carriers. This research encompassed the evaluation of physicochemical parameters (moisture content, water activity, and color characteristics), polyphenol profiling and quantification by LC-MS/MS and HPLC-DAD, determination of antioxidant capacity (Folin–Ciocalteu, TEAC ABTS, FRAP), and analysis of the in vitro dissolution behavior of polyphenolic compounds from hard gelatin capsules. The goal was to identify the most promising formulation with potential application in dietary supplements and potential for functional foods, as well as to assess the feasibility of scaling up the process to pilot-scale production.

## 2. Materials and Methods

### 2.1. Reagents

Gallic acid, chlorogenic acid, and the Folin–Ciocalteu reagent were sourced from Sigma-Aldrich (Darmstadt, Germany). Sodium carbonate was obtained from Avantor Chemicals (Gliwice, Poland). ABTS (2,2′-azino-bis(3-ethylbenzothiazoline-6-sulfonic acid)) and potassium persulfate were provided by Merck (Darmstadt, Germany). Acetonitrile of hypergrade quality for HPLC-MS/MS analyses was supplied by Merck (Darmstadt, Germany), while HPLC-MS/MS grade formic acid was purchased from VWR (Darmstadt, Germany). The hydrochloric acid used to prepare the 0.1 M solution for the polyphenol release study was purchased from Merck (Darmstadt, Germany). Deionized water (Hydrolab HLP5, Straszyn, Poland; ≤4.3 µS/cm) was used for sample preparation, chromatographic procedures, and in vitro polyphenol release studies.

### 2.2. Materials

Approximately 10 kg of dried *Ginkgo biloba* L. leaves were acquired from HerbaNordPol-Gdańsk Sp. z o.o. (Gdańsk, Poland) in August 2022 and stored under ambient conditions. Additionally, around 2 kg of dried *Clitoria ternatea* L. flowers were sourced from Barbara Angielczyk Herbal Place (Grodzisk, Poland) in August 2022 and stored under ambient conditions. Previous studies have demonstrated that the polyphenolics profile of dried leaves and flowers of both *Ginkgo biloba* L. and *Clitoria ternatea* L. was comparable to that of fresh materials, indicating that drying does not substantially alter their flavonoid or anthocyanin composition [16,17,18,19]. Therefore, dried plant materials were used in this study. Food-grade maltodextrin (Pepees S.A., Łomża, Poland) and inulin (Beneo-Orafti, Oreye, Belgium) were employed as carrier agents during both freeze-drying and spray drying processes. Gelatin capsules of size “0” were purchased from Proton Labs (Gdańsk, Poland).

### 2.3. Extraction

Five extracts were prepared for the study: *Clitoria ternatea* L., *Ginkgo biloba* L., and three mixes of these raw materials in the proportions of: 1:1, 1:2, and 2:1 (*w*/*w*) (*Clitoria ternatea* L.:*Ginkgo biloba* L.).

The extraction was performed using dried *Ginkgo biloba* L. leaves and dried *Clitoria ternatea* L. flowers, which were combined with distilled water at 95–100 °C, at a ratio of 1:20 (raw material: distilled water) (established experimentally). The mixture was maintained at this temperature for 1 h using a Thermomix (Vorewerk, Germany) with continuous stirring at 300 rpm. The pH of all extracts was within the range of 6.0–7.0. After extraction, the liquid was filtered through a nylon filter, and carriers (maltodextrin and inulin) were added at a concentration of 10% (*w*/*w*). The use of theabove mentioned carriers’ content in liquid feed was considered appropriate, as previous studies demonstrated that such concentrations provide a rational balance between encapsulation efficiency, powder yield, and preservation of bioactive compounds during spray drying, while maintaining low viscosity and good solubility of the feed mixture [20,21]. The carriers were dissolved directly in the extract under continuous stirring to ensure complete solubilization. The prepared extracts were then subjected to freeze- and spray drying. Based on the results obtained, one formulation—*Clitoria ternatea* L.:*Ginkgo biloba* L. in a 1:1 ratio with the addition of inulin, was selected for pilot-scale spray drying.

### 2.4. Drying

Freeze-drying (FD) of the liquid formulations was performed using a FreeZone 18 L system (Labconco Inc., Kansas City, MO, USA). The process was conducted under vacuum conditions (65 Pa), with the chamber maintained at −60 °C and the heating shelf set to 25 °C, over a period of 24 h. Each sample was processed in duplicate (*n =* 2).

Spray drying (SD) was conducted using a B-290 mini spray dryer (Buchi, Flawil, Switzerland). The inlet air temperature was maintained at 170 °C, while the outlet air temperature was 102 ± 1 °C. The extract—carrier mixes entered the system at 23 °C, with a feed rate of 4 mL/min and an air flow rate of 37 m^3^/h. This procedure was also carried out in duplicate (*n =* 2).

A pilot-scale spray dryer (APV ANHYDRO LAB1, Østmarken, Søborg, Copenhagen, Denmark) was also employed for the drying process. The inlet and outlet air temperatures were maintained at 170 ± 2 °C and 84 ± 2 °C, respectively, with a constant feed rate of 1.5 L/h.

The resulting powder samples were vacuum-packed and stored at −20 °C until further analysis.

### 2.5. Physical Characterization of the Powders

#### 2.5.1. Moisture Content (Mc)

Moisture content was analyzed in duplicate (*n* = 2) using the vacuum oven method, following the procedure outlined by Figiel [22]. Samples were subjected to drying at 80 °C under reduced pressure for 24 h. Results are presented as a percentage on a wet basis.

#### 2.5.2. Water Activity (*a_w_*)

Water activity was measured at 25 °C in duplicate (*n* = 2) using a dew point water activity analyzer (Model 4TE, AQUA LAB, Pullman, WA, USA).

#### 2.5.3. Color Parameters, Browning Index (BI), and Yellowness Index (YI)

Color attributes of the powdered materials were evaluated in quadruplicate (*n* = 4) using a Minolta Chroma Meter CM-700d (portable spectrophotometer) (Minolta Co., Ltd., Osaka, Japan), applying the CIE L*a*b* system. The browning index (BI) was calculated according to the formula introduced by Palou et al. [23] and Rhim et al. [24], while the yellowness index (YI) was determined following the approach proposed by Rhim et al. [24].

BI = (100 × (x − 0.31))/0.172(1)

where

x = (a* + 1.75L*)/(5.645L* + a* − 3.012b*) (2)

YI = (142.86 × b*)/L*

#### 2.5.4. Particle Size Distribution

The particle size distribution of the samples was determined with a Malvern Mastersizer 2000 laser diffraction analyzer (Malvern Panalytical, Malvern, UK) equipped for liquid measurements. Isopropanol was used as the dispersing medium to prevent dissolution of the tested material. Instrument parameters were set according to the manufacturer’s guidelines, including an absorption coefficient of 0.1 and a refractive index of 1.39 for the glass/isopropanol interface. Each sample was measured in duplicate (*n* = 2).

### 2.6. Chemical Characterization

#### 2.6.1. Qualitative and Quantitative Evaluation of Polyphenols in *Ginkgo biloba* L. and *Clitoria ternatea* L. powders *via* HPLC-MS/MS

The profiling of polyphenolic compounds was performed using high-performance liquid chromatography coupled with diode array detection (HPLC-DAD) on a Shimadzu system (Kyoto, Japan), equipped with a Luna Omega C_18_ column (1.6 µm, 100 × 2.1 mm, 100 Å; Phenomenex, Torrance, CA, USA). Separation was achieved using a gradient elution protocol with two mobile phases: phase A (1% formic acid in water, *v*/*v*) and phase B (1% formic acid in acetonitrile, *v*/*v*), delivered at 0.3 mL/min. The programmed gradient was as follows: 0–15 min, 95:5 A:B; 15–55 min, 80:20 A:B; 55–62 min, 60:40 A:B; 62.5–70 min, return to 95:5 A:B. The injection volume was 5 µL, with the column thermostat set at 30 °C and the autosampler maintained at 15 °C. DAD spectra were recorded across 190–700 nm, depending on compound class. Samples were prepared by dissolving 1 g of powder in 100 mL of a diluent composed of 95% phase A and 5% phase B (*v*/*v*), followed by sonication for 3 min and centrifugation at 3226 rcf for 5 min at 25 °C. A 1.5 mL aliquot of the supernatant was used for analysis. Quantification was carried out using standard solutions of chlorogenic acid, prepared at a final concentration of 10 µg/mL in the same diluent. Data were processed using proprietary software, and results are expressed as milligrams per gram of dry matter (mg/g DM). All analyses were carried out in duplicate (*n* = 2).

To further explore the polyphenolic profile, LC-MS/MS analysis was performed using a Shimadzu LCMS-8040 system operated with LabSolutions software (ver. 5.75). The chromatographic conditions (mobile phases, column, and gradient) were identical to those described for the DAD analysis. Mass spectrometric detection was carried out in SCAN mode across a range of 100–1500 *m*/*z*, utilizing both positive (ESI^+^) and negative (ESI^−^) electrospray ionization modes with an ionization voltage of 4.5 kV. Nitrogen (purity 99.0%, *v*/*v*) generated in-house was employed as both nebulizing (3 L/min) and drying gas (8 L/min). The interface and desolvation line were set at 250 °C and 400 °C, respectively, and data acquisition occurred at a scan rate of 1034 u/sec. Identification was supported by comparing the obtained *w*/*w* values of molecular ions (M^+^ and M^−^) and their corresponding fragment ions (PRIS(+) and PRIS(−) scans) to those reported in the literature [25,26,27,28]. Fragmentation was achieved using collision-induced dissociation (CID) with argon (purity 99.9999%, *v*/*v*) and varying collision energies (15, 35, 45, and 50 eV). Each sample was measured in duplicate (*n* = 2).

#### 2.6.2. Reducing Potential

The extraction procedure was based on the method described by Michalska-Ciechanowska et al. [29]. The reducing capacity of aqueous extracts obtained from *Ginkgo biloba* L. and *Clitoria ternatea* L. powders, formulated with carrier substances, was evaluated using the Folin–Ciocalteu colorimetric assay, as outlined by Gao et al. [30] and modified by Horszwald et al. [31]. In this method, the sample interacts with the Folin–Ciocalteu reagent under alkaline conditions, resulting in the formation of a blue complex, the intensity of which was measured spectrophotometrically at 750 nm. The absorbance readings were taken using a Synergy H1 microplate reader (BioTek Instruments Inc., Santa Clara, CA, USA). The results, obtained in duplicate (*n* = 2), are expressed as gallic acid equivalents (GAE) per 100 g of dry matter (DM) for powdered samples and as GAE per 100 mL for liquid extracts. 

#### 2.6.3. In Vitro Antioxidant Capacity

The antioxidant properties of the powders derived from *Ginkgo biloba* L. and *Clitoria ternatea* L. were assessed using two different assays: TEAC ABTS and FRAP, in accordance with the methodologies described by Re et al. [32] and Benzie and Strain [33], respectively. The TEAC ABTS method evaluates the capacity of antioxidant compounds to quench the ABTS^•+^ radical cation, whereas the FRAP assay determines the ability to reduce the Fe(III)-TPTZ complex to its ferrous form (Fe(II)-TPTZ) under acidic conditions. All analyses were performed in duplicate (*n =* 2), and the antioxidant potential was expressed as millimoles of Trolox equivalents (TE) per 100 g of dry matter (DM).

### 2.7. Encapsulation and In Vitro Release Profile of Polyphenols from Gelatin Capsules

Encapsulation was performed using a manual encapsulator manufactured by Eprus (Bielsko-Biała, Poland). The release study of polyphenolic compounds from hard gelatin capsules was carried out according to the protocol described in the European Pharmacopoeia (10th ed.), using the paddle method (apparatus II) from Electrolab (Gloucestershire, Great Britain). The test was conducted in a 0.1 M hydrochloric acid solution (pH 1.2) at 37 ± 0.5 °C with a paddle rotation speed of 75 rpm.

Each capsule was placed inside a stainless-steel sinker to prevent floating and introduced into the dissolution vessel. At predetermined time intervals (5, 10, 15 and 30 min), 5 mL aliquots were withdrawn and immediately filtered. The concentration of released polyphenols was determined by the HPLC method.

### 2.8. Statistical Analysis

Statistical evaluation of the data was conducted using STATISTICA 13 software (StatSoft, Tulsa, OK, USA). The results were expressed as mean values with corresponding standard deviations. One-way analysis of variance (ANOVA) was used to determine significant differences, followed by Tukey’s Honestly Significant Difference (HSD) test for post hoc comparisons, with statistical significance set at *p* < 0.05.

## 3. Results

### 3.1. Extraction of Ginkgo biloba L. and Clitoria ternatea L.

**Figure 1:**
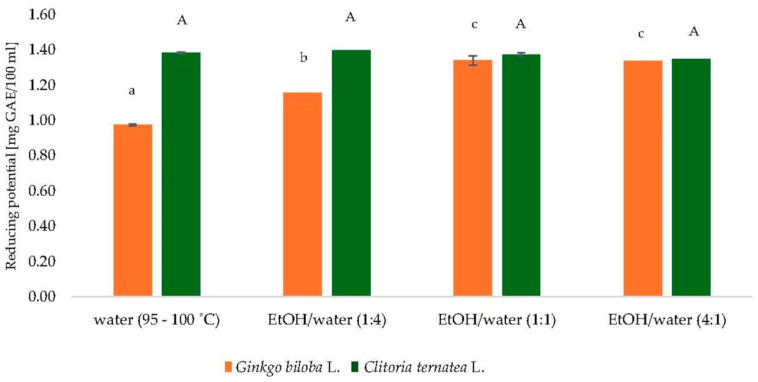
Comparison of extractants of dry leaves of *Ginkgo biloba* L. and dry flowers of *Clitoria ternatea* L. GAE—gallic acid equivalents; EtOH—ethanol; ^a–c^, mean values followed by the same letter are not statistically significantly different (*p* < 0.05) (ANOVA, Tukey’s HSD test) for *Ginkgo biloba* L. samples; ^A^ mean values followed by the same letter are not statistically significantly different (*p* < 0.05) (ANOVA, Tukey’s HSD test) for *Clitoria ternatea* L. samples.

To determine the most suitable solvent for extracting polyphenolic compounds from *Ginkgo biloba* L. and *Clitoria ternatea* L. powders, several solvent systems were assessed, including water (95–100 °C) and ethanol–water mixtures in volume ratios of 1:4, 1:1, and 4:1 (*v*/*v*). As illustrated in Figure 1, the reducing potential, expressed as gallic acid equivalents (GAE) per 100 mL, varied depending on the solvent system and botanical source.

The ethanol–water mixture at a 1:1 ratio yielded the highest reducing potential values for both plant materials, reflecting the efficiency of medium-polarity solvents in solubilizing a broad range of phenolic compounds. Although hot water (95–100 °C) resulted in slightly lower extraction yields, the differences were not substantial.

Despite the higher efficiency of hydroethanolic mixtures, hot water was ultimately chosen as the extraction solvent for subsequent analyses. This decision was guided by considerations of environmental safety, industrial applicability, and simplicity of the extraction procedure. Elevated temperature supports the release of bioactive compounds while maintaining alignment with green chemistry principles.

Given that the study encompassed both individual plant powders and their mixtures, adopting a single standardized extraction approach was essential. Therefore, water at 95–100 °C was selected as the final solvent, ensuring methodological consistency and sustainability across all samples.

### 3.2. Physical Properties of Powders

#### 3.2.1. Moisture Content

As shown in Table 1, the moisture content of the powders differed significantly (Tukey’s HSD test, *p* < 0.05) with plant material (*Ginkgo biloba* L., *Clitoria ternatea* L. and their blends at ratios 1:1, 1:2 and 2:1; *v/v*), carrier type and drying technique. Spray-dried powders had approximately 30–60% less moisture than the corresponding freeze-dried samples and 40–70% less than the controls without carrier. Within each drying technique, formulations with maltodextrin or inulin exhibited the lowest moisture contents, while the highest values occurred in carrier-free controls. Thus, both the processing method and formulation composition affected the moisture content of the final powders.

#### 3.2.2. Water Activity

As shown in Table 1, water activity of the powders differed significantly with drying technique, plant material (*Ginkgo biloba* L., *Clitoria ternatea* L., and their blends at ratios 1:1, 1:2, and 2:1), and carrier type (Tukey’s HSD test, *p* < 0.05). Spray-dried formulations exhibited approximately 50–80% lower water activity than the corresponding freeze-dried samples and up to fivefold lower values than the carrier-free controls. Within each drying technique, the lowest water activity was consistently observed in powders containing maltodextrin or inulin, whereas the highest values occurred in freeze-dried controls without carrier. These results indicate that both the processing method and the formulation composition affected the water activity of the powders. A strong positive correlation (*r* = 0.93) between moisture content and water activity was observed across all samples.

#### 3.2.3. Color, Browning Index (BI), Yellowness Index (YI)

Color is one of the key sensory and quality parameters in the evaluation of plant materials, and its instrumental measurement enables an objective assessment of the impact of processing on the visual attributes of the product. The color components in the CIE L*a*b* space (lightness, red/green and yellow/blue coordinates, accordingly), along with the browning index (BI) and yellowness index (YI), are valuable tools for characterizing the physicochemical properties of herbal extracts, offering insight into their stability and visual appeal.

As shown in Table 1, color parameters of the powders varied significantly with plant material (*Ginkgo biloba* L., *Clitoria ternatea* L. and their blends at ratios 1:1, 1:2 and 2:1), carrier type and drying technique (Tukey’s HSD test, *p* < 0.05). In general, spray-dried powders exhibited lightness (L*) values 20–50% higher than the corresponding controls and 10–30% higher than freeze-dried powders. Concomitantly, a* and b* values tended to decrease, indicating less intense red and yellow hues relative to controls. Browning index and yellowness index were reduced by approximately 30–70% in spray-dried samples compared with control powders and by 20–50% compared with freeze-dried counterparts. For all plant materials and blends, the lowest BI and YI values were recorded in formulations containing maltodextrin or inulin, whereas the highest values occurred in carrier-free controls.

In summary, formulations containing *Clitoria ternatea* L. exhibited a strong blue color due to the presence of natural anthocyanins. This effect was most pronounced in control samples without carriers, while the use of maltodextrin or inulin contributed to color stabilization and reduced pigment degradation during drying. Spray drying also appeared more effective in preserving color characteristics than freeze-drying.

### 3.3. Chemical Properties

#### 3.3.1. Qualitative Determination of Polyphenols in *Clitoria ternatea* L. and *Ginkgo biloba* L. Powders

**Figure 2:**
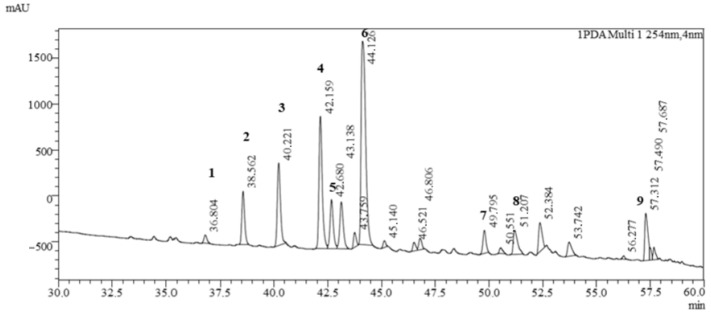
Chromatogram of *Clitoria ternatea* L. powder analyzed by UHPLC-MS/MS. 1—Myricetin 3-rutinoside; 2—Delphinidin-3-(6″-*p*-coumaroyl)-rutinoside; 3—Delphinidin-3-(cis-*p*-coumaroyl-glucoside); 4—Cyanidin-3-(6″-*p*-coumaroyl)-rutinoside; 5—Delphinidin-3-(trans-*p*-coumaroyl-glucoside); 6—Cyanidin-3-(*p*-coumaroyl)-glucoside; 7—Kaempferol 3-*O*-(2″-*O*-α-rhamnosyl-6″-*O*-malonyl)-β-glucoside; 8—Ternatin B2; 9—Ternatin D1.

The LC-MS analysis showed a diverse profile of flavonoid-type phenolic compounds (Figure 2, Table 2) of *Clitoria ternatea* L. Several flavonol glycosides were identified, including rutinosides and malonylated rhamnosyl-glucosides of myricetin and kaempferol. These compounds indicate the presence of highly glycosylated flavonols that are typical secondary metabolites of *Clitoria ternatea* L. flowers and contribute to its antioxidant capacity [13].

In addition, a rich set of anthocyanins was detected. Besides the common delphinidin and cyanidin glycosides, both cis- and trans-*p*-coumaroyl derivatives were present, demonstrating structural diversity in acylated anthocyanins. More complex ternatins, the polyacylated delphinidin derivatives characteristic of *Clitoria ternatea* L. flowers, were also found, confirming the plant’s unique pigment pattern. The detection of these ternatin molecules, together with the simpler anthocyanins, highlights the extensive modification (glycosylation and acylation) of the anthocyanin core, which is known to enhance pigment stability and color intensity [13].

Overall, the qualitative profile shows that *Clitoria ternatea* L. is particularly rich in polyacylated delphinidin-based anthocyanins alongside flavonol glycosides. This chemical fingerprint is consistent with the blue-purple color of the flowers and underpins their strong antioxidant and potential health-promoting properties.

**Figure 3:**
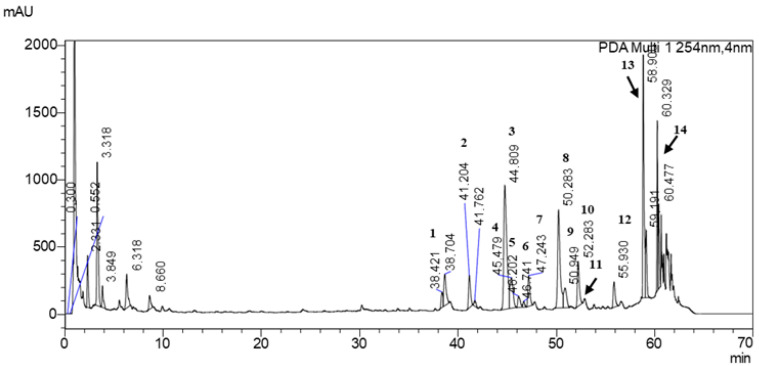
Chromatogram of *Ginkgo biloba* L. powder analyzed by UHPLC-MS/MS. 1—Myricetin 3-*O*-rutinoside; 2—Quercetin 3-*O*-[2″-(6″-*p*-coumaroyl)glucosyl]rhamnoside; 3—Quercetin 3-rutinoside; 4—Kaempferol 3-*O*-2″,6″-dirhamnosylglucoside; 5—Isorhamnetin 3-*O*-2″,6″-dirhamnosylglucoside; 6—Patuletin 3-rutinoside; 7—Patuletin 3-neohesperidoside; 8—Kaempferol 3-rutinoside; 9—Quercetin 2″-glucosylrhamnoside; 10—Kaempferol analog 1; 11—Quercetin analog 1; 12—Kaempferol analog 2; 13—Quercetin analog 2; 14—Kaempferol analog 3.

The LC-MS/MS analysis of the extract shows that its polyphenolic fraction is dominated by flavonoid glycosides, which is consistent with the well-known chemical signature of *Ginkgo biloba* L. leaves (Figure 3, Table 3) [34]. Quercetin, kaempferol, isorhamnetin and patuletin derivatives were detected, most of them occurring as rutinosides or as more complex dirhamnosyl-glucosides. The presence of both simple rutinosides and more extensively substituted glycosides indicates a broad range of glycosylation patterns typical for *Ginkgo biloba* L.

Besides the quercetin and kaempferol glycosides, several analogs and less common flavonols were tentatively identified, reflecting structural diversity in the flavonoid pool. Acylated and highly glycosylated forms were also observed, which may influence both the solubility and the pharmacokinetic behavior of these constituents. In addition to the compounds listed, several minor peaks were detected that could not be unequivocally assigned on the basis of the available MS/MS data, indicating the presence of additional, as yet unidentified phenolic constituents.

Overall, the qualitative fingerprint confirms that *Ginkgo biloba* L. contains a complex mixture of flavonoid glycosides rather than free aglycones, with quercetin, kaempferol, isorhamnetin and patuletin derivatives being the main representatives. This profile is in line with the plant’s established use as a source of antioxidant and vasomodulatory compounds and underpins its characteristic bioactivity.

#### 3.3.2. Quantitative Determination of Polyphenols in *Clitoria ternatea* L. and *Ginkgo biloba* L. Powders

The comparison of freeze-dried (FD) and spray-dried (SD) samples shows that the total content of identified polyphenols was broadly comparable between both drying techniques when the same carrier was used (Table 4). When expressed as a percentage of the FD value, the SD samples with maltodextrin showed only a slight increase in the total polyphenol content (about 4%), whereas SD samples with inulin remained at a similar level to the FD counterpart. None of these overall differences reached statistical significance, as indicated by the shared superscript letters.

For individual compounds, most anthocyanins such as delphinidin-3-(cis/trans-*p*-coumaroyl-glucoside) or ternatin derivatives were present at almost the same concentrations when FD and SD were applied, with variations typically within ±5% and no significant differences between carriers. Only a few compounds showed noticeable but still modest shifts: for example, the content of cyanidin-3-(6″-*p*-coumaroyl)-rutinoside and cyanidin-3-(*p*-coumaroyl)-glucoside was slightly (approx. 5–8%) higher in the spray-dried maltodextrin sample compared to the freeze-dried equivalent, and these differences were statistically significant.

The unidentified polyphenol fraction displayed the largest relative changes. In particular, the inulin-based SD sample contained a markedly higher amount of this fraction (over 40% more than the FD inulin sample), which was also statistically significant. In contrast, the maltodextrin-based SD and FD samples differed little in this respect.

Overall, the data indicate that the drying technique itself had only a minor effect on the content of the major identified anthocyanins and flavonol glycosides in *Clitoria ternatea* L. Statistically significant differences appeared mainly for the unidentified polyphenol fraction and for some acylated anthocyanins in specific carrier combinations. These results suggest that both carriers effectively protect the main pigments during drying, but inulin may lead to a higher content of polyphenols under spray drying conditions.

Across all drying conditions the sum of quantified flavonoids in the *Ginkgo biloba* L. powders remained in a fairly narrow range (Table 5). Expressed as a percentage of the freeze-dried values, the spray-dried samples with maltodextrin showed roughly a 5–7% higher total content of the monitored flavonol glycosides, whereas the inulin-based spray-dried material was essentially unchanged compared with its freeze-dried counterpart. According to the superscript letters these overall differences were not statistically significant.

Looking at individual compounds, most quercetin, kaempferol and isorhamnetin glycosides in the *Ginkgo biloba* L. powders exhibited only minor fluctuations between drying techniques and carriers, typically within ± 5%. Quercetin 3-rutinoside and quercetin analog 1 showed slightly higher levels in the spray-dried maltodextrin sample than in the corresponding freeze-dried sample (an increase of about 10%), and these increases reached statistical significance as indicated by the different letters. Kaempferol rutinoside and the two kaempferol analogs also displayed modest increases (concentration 10–15%) in the spray-dried variants, but with overlapping significance letters, suggesting that the effect of drying on these compounds is small.

Patuletin and isorhamnetin glycosides were largely stable across all variants of *Ginkgo biloba* L. powders, with variations within analytical error and no significant differences between carriers or drying technique. The fraction of “unidentified polyphenols” was constant in all samples, confirming that neither the choice of carrier nor the drying method substantially affected this pool.

Overall, the quantitative fingerprint indicates that the main flavonol glycosides characteristic of *Ginkgo biloba* L. is well preserved under both freeze- and spray drying conditions, with only small, compound-specific increases in the maltodextrin-based spray-dried material. Statistically significant differences were observed for only a few quercetin derivatives, while the majority of the flavonoid fraction remained unaffected by processing.

#### 3.3.3. Reducing Potential

Among powders produced with carriers, *Clitoria ternatea* L. and *Ginkgo biloba* L. blends generally exhibited higher reducing potential than single-plant powders. In the (1:2) blend the reducing potential was approximately 80–90% higher than in the corresponding single-plant powders, irrespective of drying technique (Table 6). Spray-dried and freeze-dried variants with carriers differed only slightly: changes were within 5–10% and mostly not statistically significant according to the superscript letters. Between carriers, inulin occasionally yielded 5–10% higher values than maltodextrin, but these differences were also non-significant.

The higher reducing potential observed in the blends compared with the individual plant powders suggests a possible additive or synergistic contribution of polyphenols from *Clitoria ternatea* L. and *Ginkgo biloba* L.

Control powders (without carrier) displayed much higher reducing potential than all carrier-containing samples (approximately 5–10-fold for *Ginkgo biloba* L. and 7–8-fold for *Clitoria ternatea* L.), reflecting the absence of dilution by the carrier (Table 6). Within the controls, the effect of drying technique was smaller: spray-dried controls were only about 10–15% lower than their freeze-dried counterparts for *Ginkgo biloba* L., while for the mixtures the differences were within analytical error and mostly not significant.

#### 3.3.4. Antioxidant Capacity In Vitro

For carrier-containing powders, TEAC ABTS and FRAP values followed a similar pattern. Blends of *Clitoria ternatea* L. and *Ginkgo biloba* L. showed TEAC ABTS and FRAP activities approximately 50–150% higher than the single-plant powders (Table 6). In particular, the 1:1 blend displayed intermediate to high values between the 1:2 and 2:1 mixtures and consistently higher antioxidant capacity than either plant alone. Spray-dried samples were typically 5–10% lower in TEAC ABTS and FRAP than the corresponding freeze-dried samples, but these decreases were mostly not statistically significant. Differences between maltodextrin and inulin were small (usually <10%) and significant only in a few cases.

Control powders without carrier had TEAC ABTS and FRAP values several-fold higher than the carrier-containing powders (for *Ginkgo biloba* L. about 8–10-fold higher, for *Clitoria ternatea* L. about 6–7-fold higher, depending on drying technique applied). Spray-dried controls showed TEAC ABTS values 10–15% lower than the freeze-dried controls for the same plant material, whereas FRAP values were comparable and differences were mostly not significant.

### 3.4. Evaluation of Functional Properties of the Selected 1:1 Blend of Ginkgo biloba L.:Clitoria ternatea L. Spray Drying with Inulin at Pilot Scale

#### 3.4.1. Water Activity, Reducing Potential and Antioxidant Capacity

As shown in Table 7, the *Ginkgo biloba* L.:*Clitoria ternatea* L. 1:1 blend spray-dried with inulin exhibited clear differences between the laboratory- and semi-technical-scale processes. Water activity in the semi-technical-scale powder was about 33% lower than in the laboratory-scale powder, a statistically significant reduction indicating lower water availability and potentially greater microbiological stability. Reducing potential also decreased by approximately 30% in the semi-technical-scale sample compared with the laboratory-scale sample, and this difference was statistically significant.

In contrast, antioxidant capacity measured by the TEAC ABTS assay increased by almost 90% in the semi-technical-scale powder relative to the laboratory-scale powder, which was a statistically significant improvement. FRAP values differed by about 15% between the two scales, but this change was not statistically significant.

Overall, the data in Table 7 show that scaling up from laboratory to semi-technical conditions significantly reduced water activity and reducing potential while simultaneously enhancing radical-scavenging capacity in the TEAC ABTS assay, without a significant effect on FRAP response. These trends likely reflect differences in drying dynamics and particle formation during larger-scale processing.

#### 3.4.2. Particle Size Distribution

**Figure 4:**
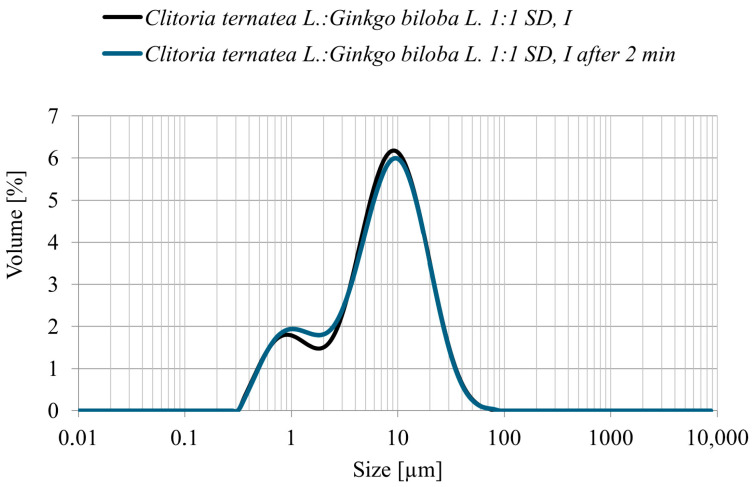
Particle size distribution (volume %) of the *Clitoria ternatea* L.: *Ginkgo biloba* L. 1:1 spray-dried powder with inulin immediately after preparation (black line) and after 2 min of measurement (blue line).

The particle size distribution (PSD) of the *Clitoria ternatea* L.:*Ginkgo biloba* L. 1:1 powder obtained by spray drying with inulin as a carrier showed a predominant fraction within 5–10 µm, with the highest frequency around 6–8 µm (Figure 4). No particles larger than 60 µm were detected, confirming the production of a fine and homogeneous powder. Measurements taken immediately after sample preparation and again after 2 min of mixing in the measurement cell yielded comparable results, indicating a stable particle size distribution and no tendency toward agglomeration. Such PSD parameters are advantageous for subsequent technological applications and may also support improved polyphenol bioavailability [35].

#### 3.4.3. Testing the Uniformity of Capsule Contents and the Release of Polyphenols from Capsules

**Figure 5:**
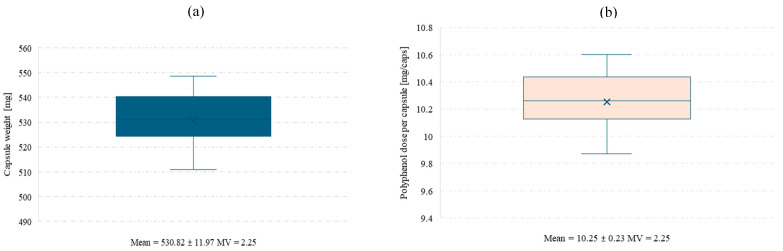
Mean capsule weight (**a**) and mean polyphenol dose per capsule (**b**) with standard deviation and coefficient of variation (MV). Low MV values confirm uniform capsule weight and polyphenol content.

In order to perform the polyphenol release study from the final product, hard gelatin capsules were prepared and filled with spray-dried powder obtained at pilot scale from an extract of *Clitoria ternatea* L. and *Ginkgo biloba* L. 1:1. For the capsule batch produced under laboratory conditions using an Eprus capsule filling machine, a uniformity of mass test was conducted for the capsules containing the *Clitoria ternatea* L.:*Ginkgo biloba* L. powder (Figure 5a,b).

**Figure 6:**
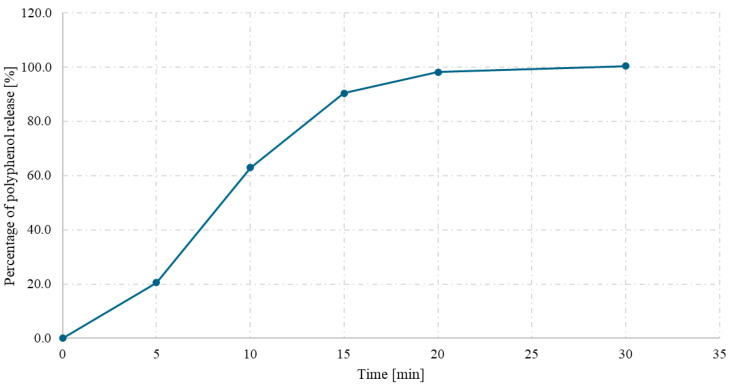
Polyphenol release (%) from the *Clitoria ternatea* L.:*Ginkgo biloba* L. 1:1 spray-dried powder with inulin as a function of time (min).

The release kinetics of polyphenols from hard gelatin capsules containing spray-dried powder of a *Ginkgo biloba* L. and *Clitoria ternatea* L. 1:1 mixture were evaluated over 45 min under simulated gastric conditions (0.1 M HCl, pH 1.2). As shown in Figure 6, a rapid release of polyphenolic compounds was observed within the first 15 min, reaching approximately 85% of the total content. After this time, the release rate began to stabilize, with more than 98% of the compounds released after 30 min.

The obtained release profile indicates efficient disintegration and dissolution of the powder encapsulated in the gelatin shell, which may be supported by the use of inulin as a carrier and the favorable physicochemical properties of the spray-dried powder. The release characteristics meet the criteria for immediate-release formulations and suggest high bioavailability of phenolic compounds under gastric conditions.

## 4. Discussion

In this study, polyphenol-rich powders were obtained from aqueous extracts of *Ginkgo biloba* L., *Clitoria ternatea* L., and their mixtures 1:1, 1:2, 2:1; *w*/*w*, by using two drying approaches, freeze-drying and spray drying, with the addition of inulin or maltodextrin as carriers. The combination of these two plant materials, representing distinct classes of polyphenolic compounds (flavonols in *Ginkgo biloba* L. leaves and anthocyanins in *Clitoria ternatea* L. flowers), enabled the evaluation of potential additive and synergistic effects in terms of reducing power, antioxidant capacity, and polyphenol release. This approach is consistent with previous reports showing that interactions among different classes of polyphenols can enhance the antioxidant capacity of products [14,15].

The use of dried plant materials was justified not only by the need to standardize the extraction process but also for practical reasons related to subsequent production stages. In industrial applications, the use of fresh raw materials would be challenging due to their seasonality, limited processing time, and higher microbiological risk. Moreover, previous studies demonstrated that the qualitative composition of polyphenols in dried *Ginkgo biloba* L. leaves and *Clitoria ternatea* L. flowers does not differ significantly from that of fresh materials, confirming the stability of their main bioactive compounds during drying [16,17,18,19]. Therefore, the use of dried materials represents a technologically and economically sound approach for the preparation of reproducible extracts.

The drying techniques significantly affected the physicochemical properties of the obtained powders. Freeze-drying, performed at low temperatures, favored the preservation of relatively high antioxidant capacity and intense color, particularly in anthocyanin-rich samples from *Clitoria ternatea* L. In contrast, spray drying, despite the higher processing temperature and shorter drying time, resulted in powders with comparable antioxidant capacity but more favorable technological parameters, including lower moisture content and reduced water activity. These results indicated that, when properly adjusted, spray drying can serve as an efficient and scalable alternative to freeze-drying for stabilizing plant extracts [36,37].

Maltodextrin and inulin, used as carriers, played a key role in stabilizing polyphenols during drying. Maltodextrin, due to its high molecular weight and ability to form homogenous encapsulating matrices, effectively limited oxidative degradation. Inulin, with its hydrophilic and porous structure, promoted the content of polyphenolic compounds [38]. The distinct effects of both carriers confirm that their selection should be adjusted to the dominant class of phenolic compounds in the extract matrix and to the desired functional properties of the final powder.

The LC-MS/MS analysis confirmed the presence of characteristic flavonol glycosides (quercetin, kaempferol, and isorhamnetin derivatives) in *Ginkgo biloba* L. samples and anthocyanins in *Clitoria ternatea* L. samples, consistent with previous literature [13,34]. No qualitative differences in polyphenolic composition were observed among the mixes, but the relative abundance of specific compounds varied depending on the ratio of raw materials. These shifts suggest possible molecular interactions between anthocyanins and flavonols, which could influence overall stability and bioactivity. The higher reducing potential and antioxidant capacity values observed in the 1:1 and 1:2 mixtures compared with individual extracts indicate additive or synergistic effects, in agreement with previous findings on inter-polyphenol interactions [14].

The polyphenol release study from gelatin capsules revealed a rapid and nearly complete release of compounds within the first 30 min, confirming high solubility and rapid disintegration of the powder matrix. The obtained dissolution profile is characteristic of immediate-release formulations and demonstrates that the drying process did not impair the bioaccessibility of the active compounds. These results suggest that the powder structure allows efficient wetting and dispersion in acidic conditions and that no strong interactions occurred between polyphenols and carriers that could delay diffusion. Similar release patterns have been described for other spray-dried polyphenolic preparations intended for dietary supplement applications [37].

The obtained powders exhibited low water activity (*a_w_* < 0.3) and moisture content below 5%, indicating potential high microbial stability and good storage performance. The color parameters correlated with anthocyanins content, and the use of inulin and maltodextrin differently influenced color stability.

Taken together, the applied drying approach and carrier selection allowed the development of powders with high antioxidant capacity, stable polyphenolic composition, and desirable technological characteristics. From a practical standpoint, the results demonstrate the feasibility of scaling up the spray drying process to semi-technical conditions, confirming its industrial potential as a cost-effective and reproducible alternative to freeze-drying. The novelty of this study lies in the combination of two phenolic-rich plant matrices with complementary compositions to achieve synergistic biological effects and in the comprehensive assessment of how drying method and carrier type affect the physicochemical stability, antioxidant capacity, and polyphenol release behavior. These findings confirm that combining *Ginkgo biloba* L. and *Clitoria ternatea* L. extracts provides an effective strategy for designing plant-based powders with targeted functional properties and application potential in dietary supplements.

## 5. Conclusions

This work highlights the role of drying technique and carrier selection in shaping the stability, antioxidant potential, and technological performance of polyphenol-rich herbal microcapsules. Spray drying, combined with inulin as a functional carrier, was identified as a cost-effective and scalable alternative to freeze-drying, ensuring good polyphenol preservation, low water activity, and a favorable particle size distribution. The synergistic 1:1 combination of *Ginkgo biloba* L. and *Clitoria ternatea* L. extracts exhibited superior antioxidant capacity and favorable release kinetics, confirming the benefits of formulating multi-plant systems. These findings provide a practical foundation for the industrial development of standardized and stable powdered products for use in dietary supplements and functional foods. Future work should focus on long-term stability assessment and in vitro bioaccessibility to further validate their application potential in nutraceutical formulations.

## Figures and Tables

**Table 1:** Selected physical properties (moisture content, water activity, color parameters, browning index—BI and yellowness index—YI) of powders obtained from *Clitoria ternatea* L., *Ginkgo biloba* L. and their blends 1:1, 1:2, 2:1 produced using different drying techniques and carrier types. SD—spray drying, FD—freeze-drying. ^a–s^—mean values followed by the same letter within the same column are not statistically significantly different (*p* < 0.05) (ANOVA, Tukey’s HSD test).

Material	Drying Technique	Carrier Type	Moisture Content [%]	Water Activity [−]	Color			BI	YI
					L*	a*	b*		
*Ginkgo biloba* L.	FD	Maltodextrin	4.88 ± 0.45 ^d^	0.1875 ± 0.0132 ^g–i^	72.99 ± 1.03 ^a^	−0.43 ± 0.01 ^r^	13.89 ± 0.13 ^c^	19.93 ± 0.07 ^c^	27.19 ± 0.09 ^c^
		Inulin	3.94 ± 0.01 ^e–g^	0.1968 ± 0.0049 ^gh^	73.45 ± 1.46 ^a^	−0.15 ± 0.04 ^pr^	13.05 ± 0.34 ^c^	18.73 ± 0.20 ^c^	25.38 ± 0.18 ^cd^
		Control	8.87 ± 0.43 ^a^	0.4197 ± 0.003 ^a^	39.05 ± 2.44 ^gh^	6.03 ± 0.25 ^a^	24.61 ± 1.20 ^a^	103.77 ± 7.10 ^a^	90.10 ± 4.19 ^a^
	SD	Maltodextrin	2.59 ± 0.11 ^j–l^	0.0843 ± 0.0032 ^pr^	72.77 ± 0.28 ^a^	−0.11 ± 0.03 ^p^	10.04 ± 0.08 ^d^	14.22 ± 0.09 ^c^	19.71 ± 0.10 ^d^
		Inulin	1.64 ± 0.09 ^mn^	0.0824 ± 0.0023 ^pr^	72.68 ± 0.32 ^a^	0.01 ± 0.01 ^p^	10.75 ± 0.03 ^d^	15.46 ± 0.01 ^c^	21.12 ± 0.03 ^cd^
		Control	2.89 ± 0.11 ^h–k^	0.2102 ± 0.0019 ^fg^	46.94 ± 0.75 ^bc^	2.95 ± 0.27 ^f–h^	17.93 ± 0.32 ^b^	51.04 ± 1.18 ^b^	54.56 ± 0.77 ^b^
*Clitoria ternatea* L.	FD	Maltodextrin	4.47 ± 0.06 ^de^	0.2374 ± 0.005 ^e^	30.7 ± 1.06 ^jk^	2.59 ± 0.02 ^i–k^	−13.14 ± 0.33 ^i–k^	−28.28 ± 0.14 ^j–n^	−61.16 ± 0.68 ^i–k^
		Inulin	4.15 ± 0.38 ^d–f^	0.2303 ± 0.004 ^ef^	37.59 ± 1.56 ^g–i^	1.46 ± 0.06 ^n^	−17.24 ± 0.53 ^rs^	−33.04 ± 0.19 ^n–p^	−65.54 ± 0.94 ^j–l^
		Control	8.86 ± 0.06 ^a^	0.3933 ± 0.001 ^b^	6.52 ± 0.48 ^p^	2.30 ± 0.50 ^kl^	−3.09 ± 0.92 ^e^	−15.28 ± 5.01 ^de^	−67.38 ± 7.19 ^kl^
	SD	Maltodextrin	2.82 ± 0.1 ^i–k^	0.1226 ± 0.0048 ^mn^	43.99 ± 1.63 ^c–f^	3.83 ± 0.1 ^d^	−14.68 ± 0.47 ^l–o^	−21.70 ± 0.08 ^e–j^	−47.68 ± 0.39 ^h^
		Inulin	1.62 ± 0.13 ^mn^	0.1045 ± 0.0079 ^n–p^	42.04 ± 0.59 ^d–g^	2.14 ± 0.03 ^lm^	−17.36 ± 0.22 ^s^	−29.36 ± 0.02 ^k–n^	−58.99 ± 0.02 ^ij^
		Control	2.28 ± 0.22 ^k–n^	0.1842 ± 0.0042 ^h–j^	23.88 ± 1.01 ^m^	4.43 ± 0.12 ^c^	−16.51 ± 0.53 ^p–s^	−38.04 ± 0.36 ^p^	−98.83 ± 1.33 ^nm^
*Clitoria ternatea* L.: *Ginkgo biloba* L. 1:1	FD	Maltodextrin	3.62 ± 0.18 ^f–h^	0.1163 ± 0.0032 ^m–o^	41.01 ± 0.81 ^e–g^	2.89 ± 0.01 ^g–i^	−14.34 ± 0.21 ^k–n^	−23.87 ± 0.01 ^f–k^	−49.96 ± 0.06 ^h^
		Inulin	3.39 ± 0.42 ^f–j^	0.1857 ± 0.0103 ^g–j^	34.86 ± 0.63 ^h–j^	2.01 ± 0.03 ^lm^	−15.57 ± 0.18 ^n–p^	−31.12 ± 0.10 ^l–o^	−63.81 ± 0.27 ^i–k^
		Control	7.09 ± 0.3 ^b^	0.3440 ± 0.001 ^c^	20.22 ± 1.00 ^mn^	3.23 ± 0.06 ^ef^	−9.09 ± 0.37 ^f^	−25.12 ± 0.46 ^g–m^	−64.18 ± 0.24 ^i–k^
	SD	Maltodextrin	2.39 ± 0.26 ^k–m^	0.1125 ± 0.0033 ^m–o^	48.28 ± 0.52 ^bc^	3.74 ± 0.04 ^d^	−12.83 ± 0.31 ^g–j^	−17.36 ± 0.04 ^d–f^	−37.97 ± 0.05 ^fg^
		Inulin	1.99 ± 0.05 ^l–n^	0.1339 ± 0.0033 ^lm^	45.25 ± 2.92 ^c–e^	2.65 ± 0.12 ^ij^	−15.92 ± 0.86 ^o–r^	−24.77 ± 0.01 ^g–l^	−50.27 ± 0.06 ^h^
		Control	2.38 ± 0.11 ^k–m^	0.1619 ± 0.0069 ^jk^	28.82 ± 0.13 ^kl^	4.62 ± 0.06 ^bc^	−14.32 ± 0.27 ^k–n^	−28.13 ± 0.13 ^i–n^	−70.99 ± 0.44 ^l^
*Clitoria ternatea* L.: *Ginkgo biloba* L. (1:2)	FD	Maltodextrin	4.49 ± 0.05 ^de^	0.1682 ± 0.0007 ^i–k^	39.78 ± 0.98 ^fg^	2.53 ± 0.01 ^jk^	−12.34 ± 0.22 ^g–i^	−21.51 ± 0.04 ^e–i^	−44.33 ± 0.33 ^gh^
		Inulin	4.00 ± 0.07 ^e–g^	0.1981 ± 0.0059 ^gh^	41.79 ± 1.17 ^d–g^	1.01 ± 0.03 ^o^	−13.09 ± 0.37 ^h–k^	−24.26 ± 0.01 ^g–k^	−44.78 ± 0.01 ^h^
		Control	7.59 ± 0.11 ^b^	0.3827 ± 0.0059 ^b^	24.59 ± 1.99 ^lm^	2.69 ± 0.17 ^h–j^	−8.62 ± 0.57 ^f^	−21.48 ± 0.21 ^e–h^	−50.08 ± 0.57 ^h^
	SD	Maltodextrin	1.93 ± 0.03 ^l–n^	0.0640 ± 0.0018 ^r^	50.82 ± 1.70 ^b^	3.07 ± 0.06 ^e–g^	−9.51 ± 0.27 ^f^	−12.32 ± 0.02 ^d^	−26.72 ± 0.04 ^e^
		Inulin	1.51 ± 0.08 ^n^	0.0967 ± 0.0045 ^op^	45.59 ± 0.69 ^cd^	1.99 ± 0.06 ^m^	−11.69 ± 0.2 ^gh^	−18.83 ± 0.04 ^d–g^	−36.63 ± 0.03 ^f^
		Control	1.62 ± 0.01 ^mn^	0.1272 ± 0.0076 ^mn^	33.81 ± 0.63 ^ij^	4.81 ± 0.19 ^b^	−11.56 ± 0.52 ^g^	−18.85 ± 0.01 ^d–g^	−48.83 ± 0.08 ^h^
*Clitoria ternatea* L.: *Ginkgo biloba* L. (2:1)	FD	Maltodextrin	3.21 ± 0.06 ^g–j^	0.0823 ± 0.003 ^pr^	33.45 ± 0.08 ^ij^	3.16 ± 0.03 ^e–g^	−13.46 ± 0.07 ^i–l^	−25.97 ± 0.05 ^h–m^	−57.49 ± 0.27 ^i^
		Inulin	3.44 ± 0.12 ^f–i^	0.1526 ± 0.0065 ^kl^	32.65 ± 0.84 ^jk^	2.21 ± 0.04 ^lm^	−15.20 ± 0.33 ^m–p^	−31.70 ± 0.12 ^m–p^	−66.52 ± 0.32 ^kl^
		Control	5.89 ± 0.09 ^c^	0.2901 ± 0.0045 ^d^	13.20 ± 0.99 ^o^	3.26 ± 0.08 ^e^	−9.46 ± 0.47 ^f^	−36.03 ± 0.26 ^op^	−102.47 ± 2.28 ^n^
	SD	Maltodextrin	2.00 ± 0.11 ^l–n^	0.0868 ± 0.0155 ^pr^	41.60 ± 0.23 ^d–g^	4.33 ± 0.18 ^c^	−14.01 ± 0.57 ^j–m^	−20.85 ± 0.15 ^e–h^	−48.09 ± 0.19 ^h^
		Inulin	1.54 ± 0.08 ^n^	0.0952 ± 0.0071 ^op^	31.78 ± 0.66 ^jk^	3.25 ± 0.07 ^e^	−14.71 ± 0.27 ^l–o^	−29.43 ± 0.16 ^k–o^	−66.11 ± 0.27 ^kl^
		Control	1.57 ± 0.14 ^n^	0.1149 ± 0.005 ^m–o^	19.27 ± 0.08 ^n^	4.74 ± 0.03 ^b^	−12.76 ± 0.06 ^g–j^	−33.07 ± 0.18 ^n–p^	−94.63 ± 0.90 ^m^

**Table 2:** Identification of polyphenol compounds in *Clitoria ternatea* L. powder using its spectral characteristics, positive and negative ions in HPLC-MS/MS.

Peak	RT (min)	*m*/*z* (Positive)	*m*/*z* (Negative)	*m*/*z* PRIS Ions (+/− Mode)	Collision Energy [eV]	Compound Name	Class
1	36.804	627	625	319	35	Myricetin 3-rutinoside	Flavonoids
2	38.562	757	755	611,303	35	Delphinidin-3-(6″-*p*-coumaroyl)-rutinoside	Anthocyanins
3	40.221	611	609	303	35	Delphinidin-3-(cis-*p*-coumaroyl-glucoside)	Anthocyanins
4	42.159	742	740	287	35	Cyanidin-3-(6″-*p*-coumaroyl)-rutinoside)	Anthocyanins
5	42.680	611	609	303	35	Delphinidin-3-(trans-*p*-coumaroyl-glucoside)	Anthocyanins
6	44.126	595	593	287	35	Cyanidin-3-(*p*-coumaroyl)-glucoside	Anthocyanins
7	49.795	681	679	287, 127	35	Kaempferol 3-*O*-(2″″-*O*-α-rhamnosyl-6″″-*O*-malonyl)-β-glucoside	Flavonoids
8	51.207	1637	−	1388,303	35	Ternatin B2	Anthocyanins
9	57.312	1785	1783	303	35	Ternatin D1	Anthocyanins

RT—retention time.

**Table 3:** Identification of polyphenol compounds in *Ginkgo biloba* L. powder using its spectral characteristics, positive and negative ions in HPLC-MS/MS.

Peak	RT (min)	*m*/*z* (Positive)	*m*/*z* (Negative)	*m*/*z* PRIS Ions (+/− Mode)	Collision Energy [eV]	Compound Name	Class
1	38.4	627	625	319, 316	35	Myricetin 3-*O*-rutinoside	Flavonoids
2	41.2	757	755	303, 300	35	Quercetin 3-*O*-[2″-(6″-*p*-coumaroyl)glucosyl]rhamnoside	Flavonoids
3	44.8	611	609	303, 300	35	Quercetin 3-rutinoside	Flavonoids
4	45.5	747	−	287	35	Kaempferol 3-*O*-2″,6″-dirhamnosylglucoside	Flavonoids
5	46.2	771	769	317, 129, 639, 605, 314, 299	35	Isorhamnetin 3-*O*-2″,6″-dirhamnosylglucoside	Flavonoids
6	46.7	−	883	769, 314, 113	35	Patuletin 3-rutinoside	Flavonoids
7	47.2	641	639	333, 330, 315	35	Patuletin 3-neohesperidoside	Flavonoids
8	50.3	595	593	287, 285	35	Kaempferol 3-rutinoside	Flavonoids
9	50.9	611	609	345, 303, 147, 129	35	Quercetin 2″-glucosylrhamnoside	Flavonoids
10	52.3	595	593	287, 285, 271	35	Kaempferol analog 1	Flavonoids
11	52.4	611	−	449, 413, 345, 303, 201	35	Quercetin analog 1	Flavonoids
12	55.9	595	593	287, 284, 271	35	Kaempferol analog 2	Flavonoids
13	58.9	871	869	757, 755, 303, 291, 257, 165, 147	35	Quercetin analog 2	Flavonoids
14	60.3	855	853	747, 739, 593, 287, 284, 257, 165, 147, 113	35	Kaempferol analog 3	Flavonoids

RT—retention time.

**Table 4:** Quantitative determination of polyphenol compounds in *Clitoria ternatea* L. powder using HPLC-DAD technique. SD—spray drying, FD—freeze-drying, DM—dry matter; ^a–b^ mean values followed by the same letter are not statistically significantly different (*p* < 0.05) (ANOVA, Tukey’s HSD test).

Compound Name	FD		SD	
	Maltodextrin	Inulin	Maltodextrin	Inulin
	[mg/g DM]			
Myricetin 3-rutinoside	0.09 ± 0.07 ^a^	0.09 ± 0.01 ^a^	0.10 ± 0.01 ^a^	0.09 ± 0.01 ^a^
Delphinidin-3-(6″-*p*-coumaroyl)-rutinoside	0.54 ± 0.02 ^b^	0.52 ± 0.01 ^b^	0.61 ± 0.02 ^a^	0.55 ± 0.01 ^ab^
Delphinidin-3-(cis-*p*-coumaroyl-glucoside)	0.98 ± 0.03 ^a^	0.90 ± 0.02 ^a^	1.02 ± 0.06 ^a^	1.04 ± 0.01 ^a^
Cyanidin-3-(6″-*p*-coumaroyl)-rutinoside)	1.61 ± 0.05 ^b^	1.57 ± 0.10 ^b^	1.75 ± 0.01 ^a^	1.63 ± 0.01 ^b^
Delphinidin-3-(trans-*p*-coumaroyl-glucoside)	0.54 ± 0.07 ^a^	0.55 ± 0.02 ^a^	0.62 ± 0.01 ^a^	0.57 ± 0.01 ^a^
Cyanidin-3-(*p*-coumaroyl)-glucoside	4.50 ± 0.02 ^b^	4.31 ± 0.06 ^b^	4.84 ± 0.14 ^a^	4.50 ± 0.06 ^b^
Kaempferol 3-*O*-(2″″-*O*-α-rhamnosyl-6″″-*O*-malonyl)-β-glucoside	0.29 ± 0.01 ^ab^	0.27 ± 0.01 ^b^	0.30 ± 0.01 ^a^	0.28 ± 0.01 ^ab^
Ternatin B2	0.38 ± 0.01 ^a^	0.08 ± 0.01 ^a^	0.40 ± 0.01 ^a^	0.37 ± 0.01 ^a^
Ternatin D1	0.55 ± 0.07 ^a^	0.51 ± 1.00 ^a^	0.59 ± 0.01 ^a^	0.55 ± 0.01 ^a^
Unidentified polyphenols	7.73 ± 0.10 ^ab^	5.58 ± 0.10 ^b^	7.66 ± 0.10 ^ab^	8.02 ± 0.10 ^a^
SUM	17.21 ± 0.36 ^a^	14.68 ± 0.52 ^b^	17.89 ± 0.95 ^a^	17.60 ± 0.33 ^a^

**Table 5:** Quantitative determination of polyphenol compounds in *Ginkgo biloba* L. powder using HPLC-DAD technique. SD—spray drying, FD—freeze drying, DM — dry matter; ^a–c^ mean values followed by the same letter are not statistically significantly different (*p* < 0.05) (ANOVA, Tukey’s HSD test).

Compound Name	FD		SD	
	Maltodextrin	Inulin	Maltodextrin	Inulin
	[mg/g DM]			
Quercetin 3-rutinoside	0.80 ± 0.05 ^ab^	0.75 ± 0.01 ^b^	0.89 ± 0.01 ^a^	0.85 ± 0.03 ^ab^
Kaempferol 3-*O*-2″-6″-dirhamnosylglucoside	0.33 ± 0.06 ^a^	0.28 ± 0.01 ^a^	0.39 ± 0.02 ^a^	0.37 ± 0.04 ^a^
Isorhamnetin 3-*O*-2″-6″-dirhamnosylglucoside	0.02 ± 0.01 ^a^	0.03 ± 0.01 ^a^	0.02 ± 0.01 ^a^	0.03 ± 0.01 ^a^
Patuletin 3-neohesperidoside	0.25 ± 0.01 ^ab^	0.24 ± 0.09 ^b^	0.27 ± 0.01 ^a^	0.27 ± 0.01 ^a^
Kaempferol 3-rutinoside	0.32 ± 0.01 ^b^	0.32 ± 0.03 ^b^	0.36 ± 0.01 ^a^	0.36 ± 0.01 ^a^
Quercetin 2″-glucosylrhamnoside	0.30 ± 0.01 ^a^	0.31 ± 0.01 ^a^	0.32 ± 0.01 ^a^	0.32 ± 0.01 ^a^
Kaempferol analog 1	0.31 ± 0.01 ^ab^	0.28 ± 0.09 ^b^	0.34 ± 0.01 ^a^	0.33 ± 0.01 ^a^
Kaempferol analog 2	0.19 ± 0.01 ^b^	0.20 ± 0.08 ^b^	0.23 ± 0.01 ^a^	0.22 ± 0.01 ^a^
Quercetin analog 1	0.97 ± 0.01 ^b^	0.95 ± 0.01 ^c^	1.03 ± 0.01 ^a^	1.02 ± 0.01 ^a^
Unidentified polyphenols	5.30 ± 0.01 ^a^	5.51 ± 0.01 ^a^	5.56 ± 0.01 ^a^	5.38 ± 0.01 ^a^
SUM	8.78 ± 0.02 ^a^	8.86 ± 0.18 ^a^	9.40 ± 0.34 ^a^	9.17 ± 5.50 ^a^

**Table 6:** Reducing potential (expressed as g gallic acid equivalents (GAE) per 100 g dry matter (DM)) and antioxidant capacity (TEAC ABTS and FRAP, expressed as mmol Trolox per 100 g dry matter) of powders obtained from *Ginkgo biloba* L., *Clitoria ternatea* L. and their blends produced using different drying techniques and carrier types and control samples (without carriers). SD—spray drying, FD—freeze drying. ^a–k^—mean values followed by the same letter are not statistically significantly different (*p* < 0.05) (ANOVA, Tukey’s HSD test) among carrier-containing samples. ^A–F^—mean values followed by the same letter are not statistically significantly different (*p* < 0.05) (ANOVA, Tukey’s HSD test) among control samples without carriers.

Material	Drying Technique	Carrier Type	Reducing Potential [g GAE/100 g DM]	TEAC ABTS [mmol Trolox/100 g DM]	FRAP [mmol Trolox/100 g DM]
*Ginkgo biloba* L.	FD	Maltodextrin	0.283 ± 0.030 ^d^	1.88 ± 0.09 ^cd^	2.19 ± 0.07 ^fg^
		Inulin	0.283 ± 0.020 ^d^	1.83 ± 0.08 ^de^	2.22 ± 0.02 ^fg^
		Control	2.824 ± 0.07 ^A^	19.07 ± 0.15 ^A^	21.82 ± 1.62 ^A^
	SD	Maltodextrin	0.286 ± 0.020 ^cd^	1.77 ± 0.06 ^d–f^	2.18 ± 0.07 ^fg^
		Inulin	0.279 ± 0.011 ^d^	1.69 ± 0.01 ^e–h^	2.10 ± 0.08 ^g^
		Control	2.421 ± 0.205 ^B^	16.08 ± 0.73 ^B^	19.59 ± 1.55 ^A^
*Clitoria ternatea* L.	FD	Maltodextrin	0.226 ± 0.023 ^d^	0.95 ± 0.02 ^k^	1.31 ± 0.06 ^h^
		Inulin	0.214 ± 0.008 ^d^	0.91 ± 0.03 ^k^	1.23 ± 0.04 ^h^
		Control	1.764 ± 0.104 ^CD^	6.65 ± 0.15 ^F^	10.08 ± 0.08 ^D^
	SD	Maltodextrin	0.218 ± 0.017 ^d^	0.84 ± 0.01 ^k^	1.27 ± 0.04 ^h^
		Inulin	0.253 ± 0.021 ^d^	0.82 ± 0.02 ^k^	1.23 ± 0.06 ^h^
		Control	1.706 ± 0.082 ^E^	5.99 ± 0.08 ^F^	9.97 ± 0.10 ^D^
*Clitoria ternatea* L.:*Ginkgo biloba* L. 1:1	FD	Maltodextrin	0.401 ± 0.006 ^ab^	1.74 ± 0.01 ^d–g^	2.60 ± 0.02 ^c^
		Inulin	0.397 ± 0.018 ^ab^	1.67 ± 0.06 ^f–h^	2.49 ± 0.04 ^c–e^
		Control	2.041 ± 0.131 ^CD^	8.53 ± 0.14 ^E^	12.22 ± 0.02 ^CD^
	SD	Maltodextrin	0.388 ± 0.047 ^ab^	1.62 ± 0.02 ^g–i^	2.50 ± 0.10 ^cd^
		Inulin	0.419 ± 0.017 ^ab^	1.57 ± 0.05 ^hi^	2.39 ± 0.04 ^c–f^
		Control	2.232 ± 0.251 ^BC^	8.05 ± 0.11 ^E^	11.64 ± 0.15 ^D^
*Clitoria ternatea* L.:*Ginkgo biloba* L. (1:2)	FD	Maltodextrin	0.432 ± 0.031 ^a^	2.21 ± 0.03 ^a^	3.26 ± 0.09 ^a^
		Inulin	0.427 ± 0.046 ^ab^	2.09 ± 0.05 ^ab^	2.97 ± 0.02 ^b^
		Control	2.250 ± 0.182 ^B–D^	11.3 ± 0.15 ^C^	15.74 ± 0.22 ^B^
	SD	Maltodextrin	0.424 ± 0.026 ^ab^	2.03 ± 0.03 ^bc^	3.04 ± 0.15 ^ab^
		Inulin	0.433 ± 0.027 ^a^	2.05 ± 0.02 ^b^	3.05 ± 0.01 ^ab^
		Control	2.322 ± 0.043 ^BC^	10.17 ± 0.08 ^D^	14.64 ± 0.24 ^BC^
*Clitoria ternatea* L.:*Ginkgo biloba* L. (2:1)	FD	Maltodextrin	0.358 ± 0.031 ^bc^	1.48 ± 0.03 ^ij^	2.37 ± 0.10 ^c–g^
		Inulin	0.375 ± 0.031 ^ab^	1.42 ± 0.01 ^j^	2.25 ± 0.01 ^d–g^
		Control	1.760 ± 0.211 ^DE^	6.79 ± 0.07 ^F^	9.93 ± 0.25 ^D^
	SD	Maltodextrin	0.371 ± 0.034 ^ab^	1.41 ± 0.05 ^j^	2.27 ± 0.01 ^d–g^
		Inulin	0.386 ± 0.014 ^ab^	1.36 ± 0.03 ^j^	2.23 ± 0.04 ^e–g^
		Control	1.89 ± 0.113 ^C–E^	6.76 ± 0.08 ^F^	9.58 ± 0.15 ^D^

**Table 7:** Water activity, reducing potential and antioxidant capacity of the *Ginkgo biloba* L.:*Clitoria ternatea* L. 1:1 powder with inulin, spray-dried (SD) at laboratory and semi-technical scale. SD—spray drying, DM—dry matter. Different superscript letters (^a,b^) within the same column indicate significant differences (*p* < 0.05) according to Tukey’s post hoc test.

Material	Drying Technique	Water Activity [−]	Reducing Potential [g GAE/100 g DM]	TEAC ABTS [mmol Trolox/100 g DM]	FRAP [mmol Trolox/100 g DM]
Laboratory scale	SD	0.1339 ± 0.0033 ^a^	0.42 ± 0.01 ^a^	1.57 ± 0.05 ^b^	2.39 ± 0.04 ^a^
Semi-technical scale		0.0896 ± 0.0013 ^b^	0.29 ± 0.01 ^b^	2.93 ± 0.26 ^a^	2.05 ± 0.12 ^a^

## Data Availability

The original contributions presented in this study are included in the article. Further inquiries can be directed to the corresponding authors.
